# Supplementary material for: Topoisomerase 3β interacts with RNAi machinery to promote heterochromatin formation and transcriptional silencing in Drosophila
Source: Nat Commun. 2018 Nov 23;9:4946. doi: 10.1038/s41467-018-07101-4 (PMC6251927; doi:10.1038/s41467-018-07101-4)
Supplement: Supplementary file 1 — Supplementary Information [file 41467_2018_7101_MOESM1_ESM.pdf]

Supplementary Information

Topoisomerase 3β Interacts with RNAi Machinery to Promote Heterochromatin Formation and Transcriptional Silencing in *Drosophila*

Lee, et al.

Supplementary Figure 1

A

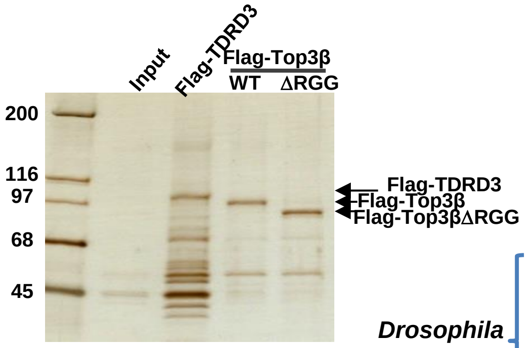

B

Identified Proteins	Mock -IP	Flag-TDRD3		Flag-Top3β		Flag-Top3β-ΔRGG	
RNase	-	-	+	-	+	-	+
TDRD3	0	1000	1295	189	248	207	198
Top3β	0	106	140	803	968	1057	1037
FMRP	0	199	5 (3%)	53	0 (0%)	31	0 (0%)
p68	0	211	15 (7%)	31	0 (0%)	11	0 (0%)
AGO2	0	7	4 (57%)	19	22 (116%)	26	27 (104%)
VIG	0	0	0	0	0	0	0

Drosophila RISC (bracket: FMRP, p68, AGO2, VIG)

C

	TDRD3-A		TDRD3-c	
RNase	-	+	-	+
TDRD3	44	45 (102%)	83	68 (82%)
Top3β	25	12 (48%)	51	36 (71%)
P68	0	0	26	2 (8%)
FMRP	9	1 (11%)	29	1 (3%)
AGO2	7	5 (71%)	32	10 (31%)
VIG	8	10 (125%)	0	0

Drosophila RISC (bracket: P68, FMRP, AGO2, VIG)

Supplementary Figure 1. The association between Top3β-TDRD3 and FMRP-containing RISC is stabilized by RNA.
(A) An image of a silver-stained SDS-PAGE gel after immunoprecipitation using a Flag antibody from S2 cells expressing Flag-TDRD3 and Flag-Top3β constructs. ΔRGG is a Top3β mutant deleted of its RNA binding domain.
(B) A table shows a select list of the number of peptides identified by MS analysis of the immunoprecipitates of Top3β and TDRD3 as shown in (A). The presence (+) or absence (-) RNase A treatment is indicated. The percentage of the number of peptides after RNase treatment in comparison to that before the treatment is shown in parentheses. The proteins that are known RISC components are indicated by a bracket.
(C) A table shows a select list of the number of peptides identified by MS analysis of the immunoprecipitate by two TDRD3 antibodies from S2 cells. See (**B**) for description.

Supplementary Figure 2

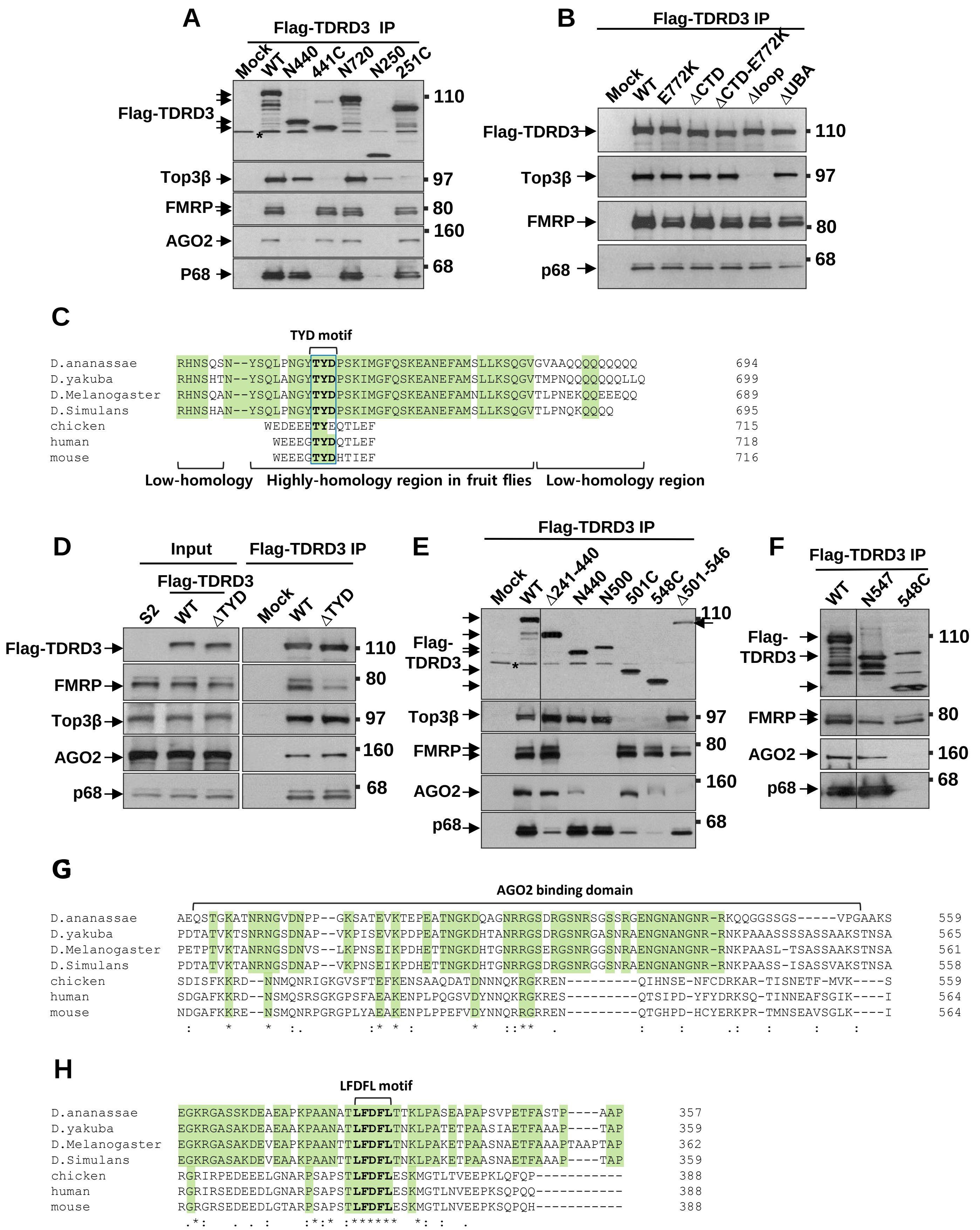

Supplementary Figure 2. TDRD3 is a scaffold that interacts with Top3β and RISC with different domains.

(A, B, D-F) IP-Western data of mapping the domains of TDRD3 that interact with Top3β and different RISC components. Flag-tagged TDRD3 is expressed in S2 cells followed by co-immunoprecipitation using a Flag antibody. The asterisks represents non-specific signals due to antibody cross reactivity.

(C, G, and H) Sequence alignments of the FMRP-binding domain which includes the TYD motif (**C**), AGO2-binding domain (**G**), and the p68-binding domain which includes LFDFL motif (H). Protein sequences from 4 *Drosophilae* (*ananassae, yakuba, melanogaster,* and *simulans*) and 3 mammals (chicken, human, and, mouse) TDRD3 are shown. The numbers on the right column represent amino acid numbers. The green color highlights conserved residues. The asterisks mark residues that are identical in all species. The double dots mark conserved residues of similar properties. The single dots mark partially conserved residues of of similar properties. Note that the LFDFL motif and the TYD motif are highly conserved in all sequences, whereas the AGO2 binding domain is conserved only in insects.

Supplementary Figure 3

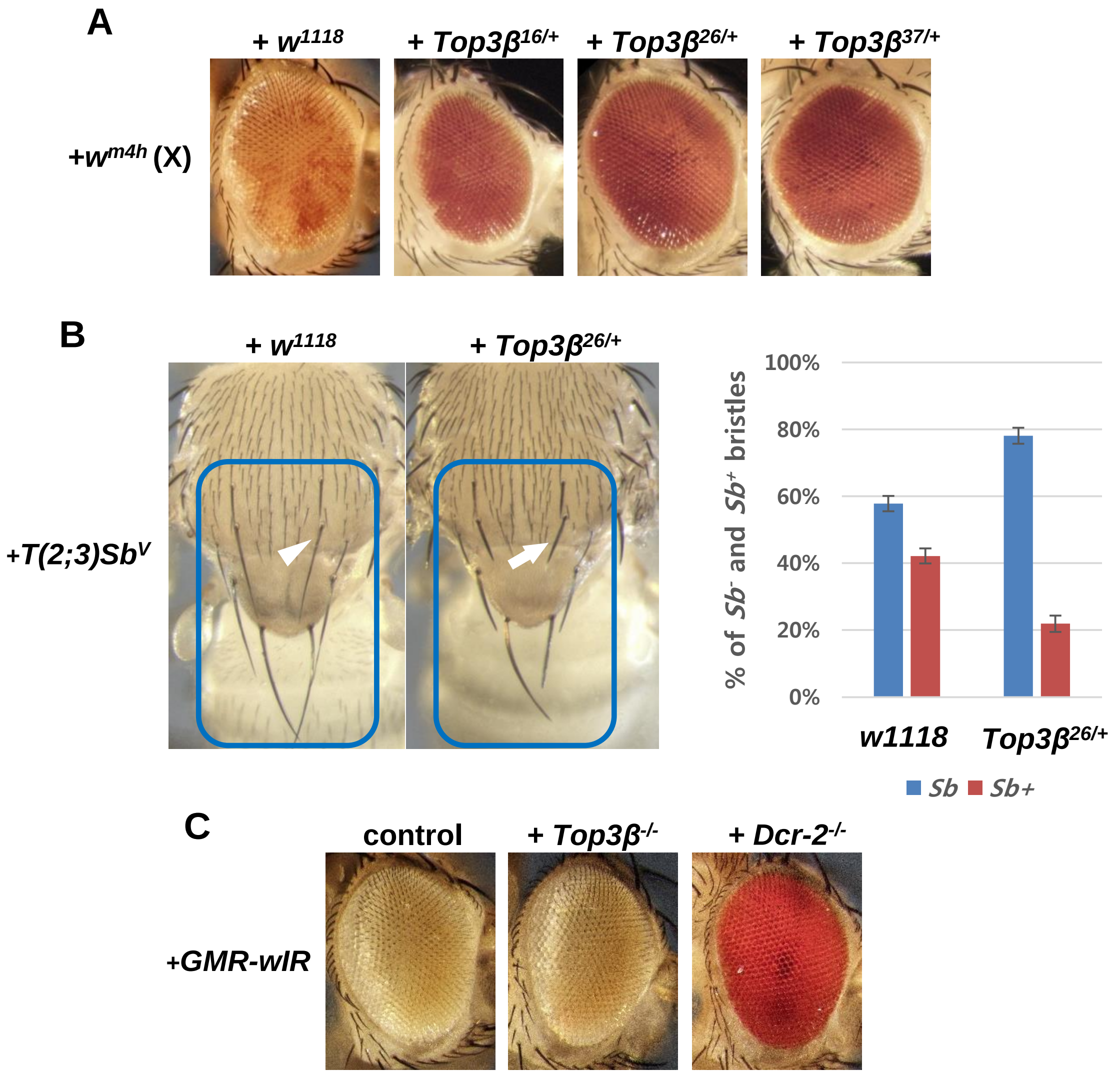

Supplementary Figure 3. *Top3β* mutation disrupts heterochromatic gene silencing in PEV assays.

(A) Representative eye images showing that w^{m4h} PEV reporter line is strongly suppressed by three different *Top3β* mutant alleles, as indicated on top. The flies in each panel carry a copy of the PEV reporter and a copy of the *Top3β* mutant allele: $Top3\beta^{16}$, $Top3\beta^{26}$, and $Top3\beta^{37}$ (Wu et al., 2006). All three alleles lack the full-length Top3β protein.

(B) Representative images (left) and quantification (right) of the bristle PEV reporter assay (Sinclair, Mottus et al. 1983) showing that $Top3\beta^{26}$ mutation suppresses the *Stubble* (Sb^1) variegation reporter $T(2;3)Sb^v$. This reporter is derived from the dominant *Sb* gene mutation which causes short bristle phenotype. The relocated Sb^1 mutant ($T(2;3)Sb^v$) displays variegated bristle phenotype with mixture of long (Sb^+: arrowhead) and short (Sb^1: arrow) bristles on 14 major bristles on the back. The blue highlights the comparison of wild type (Sb^+) v.s. shortened (*Sb*) phenotype due to repression and de-repression of Sb^v expression upon *Top3β*. The t-test values between *Top3β* and control is p=0.0004. The error bars represent standard error.

(C) Representative images showing that *Top3β* mutation does not affect post-transcriptional silencing. The flies induces *white* RNAi in the eye by transgene *GMR-wIR* (control left). *Top3β* mutant does not modify *white* RNAi effect (middle) whereas *Dcr-2* mutant suppresses white silencing (right).

Supplementary Figure 4

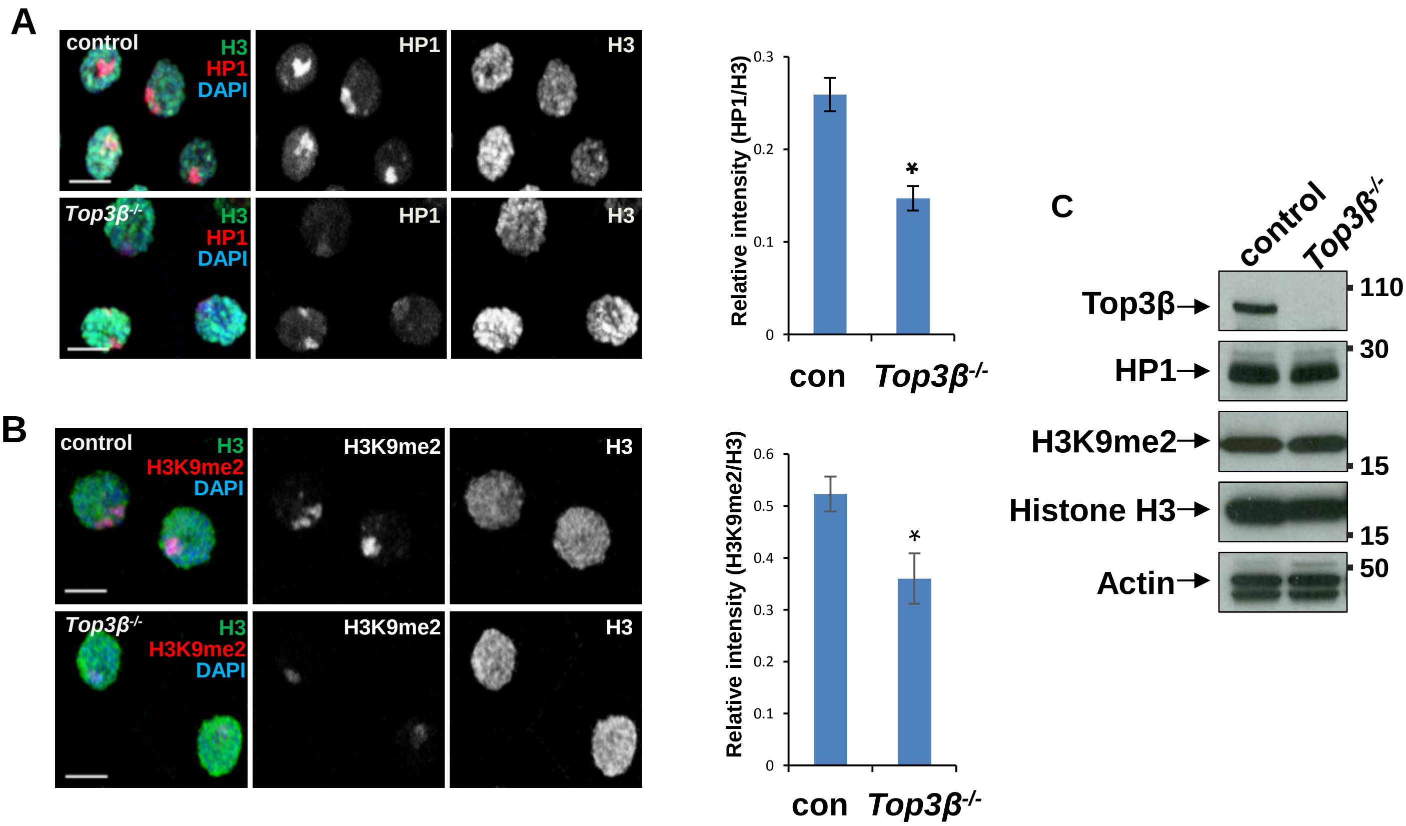

Supplementary Figure 4. ***Top3β*** **mutant displays altered cellular distributions of HP1 and H3K9me2.**

(A) Representative z-stacked confocal immunofluorescence images (left) and their quantification (right) of 3rd instar larval salivary glands show that *Top3β* mutants display altered distribution of heterochromatin HP1 (red) when compared to the control flies (w^{1118}). Histone H3 (green) and DNA (blue) were co-stained as controls. The graph on the right indicates the ratio between the signal intensity of HP1 and that of histone H3. At least 50 nuclei were analyzed for the assay. The asterisk represents $p<0.05$ by student T-test. The error bars represent standard error. The scale bars represent 10μm.

(B) Same as (**A**), except that H3K9me2 was stained.

(C) Western blotting shows similar HP1 and H3K9me2 levels in w^{1118} control and *Top3β* mutant fly head lysates.

Supplementary Figure 5

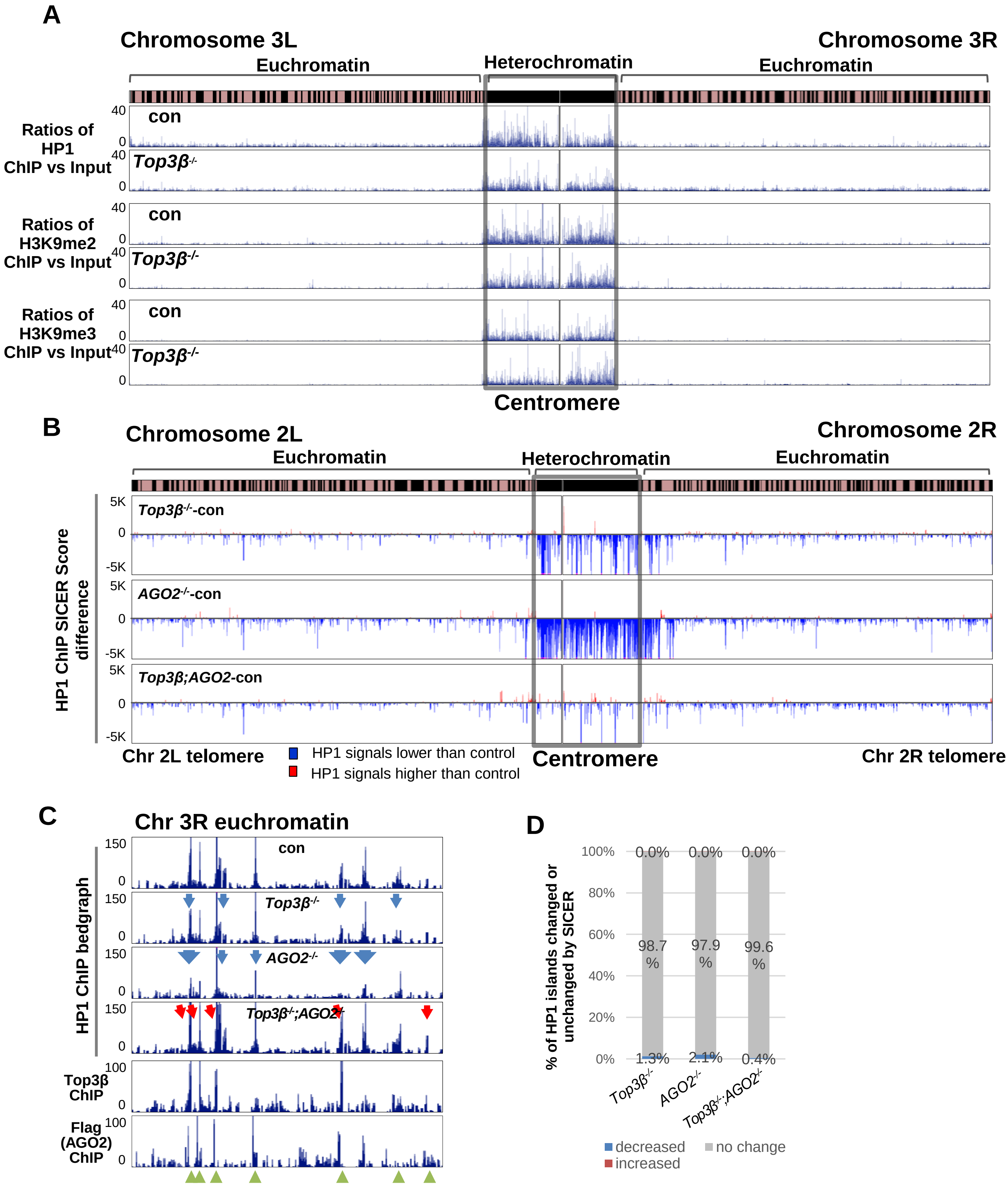

Supplementary Figure 5. *Top3β* and *AGO2* mutations disrupt HP1 distribution by ChIP-seq assays

(A) Bedgraphs show the ratio between the ChIP signals of HP1, H3K9me2, and H3K9me3 compared to their respective input signals for chromosome 3 of *w*^{1118} control and *Top3β* knockout mutants. Note that all three marks are enriched in the heterochromatin.

(B) Bedgraphs showing SICER score difference of the HP1 ChIP-seq data between *Top3β* and *AGO2* single mutants *versus* the control for chromosome 2L (left) and 2R (right). The data indicate that the HP1 level in pericentric regions of chromosome 2 is largely reduced in *Top3β* and *AGO2* single mutants, but this reduction is suppressed in the double mutant. The HP1-decreased islands (HDIs) and HP1-increased islands (HIIs) are marked by blue and red, respectively. A cut-off of 200 is applied to the data to exclude low SICER signals. False discovery rate is <5%.

(C) HP1 signals are partially reduced in euchromatin region of *Top3β* and *AGO2* single mutant, but enhanced in the double mutant. Bedgraphs of HP1 ChIP-seq data of *w*^{1118} control, *Top3β* mutant, *AGO2* mutant, and *Top3β;AGO2* double mutant from the top, followed by Top3β ChIP and Flag-ChIP to mark chromatin localization of AGO2 in control flies. The blue arrows indicate a decrease in HP1 ChIP signals and red arrows an increase in HP1 ChIP signals. The green arrowheads on the bottom represent co-localization of HP1, Top3β, and Flag-AGO2 ChIP signals.

(D) A graph shows the percentage of HP1 islands that are decreased (blue), increased (red), or of no change (gray) in the euchromatin of chromosomes 2 and 3 for each mutant when compared to the control. The quantification is based on the average SICER scores from 3 independent ChIP experiments for *Top3β*, 2 for *AGO2*, and 2 for *Top3β;AGO2* mutants.

Supplementary Figure 6

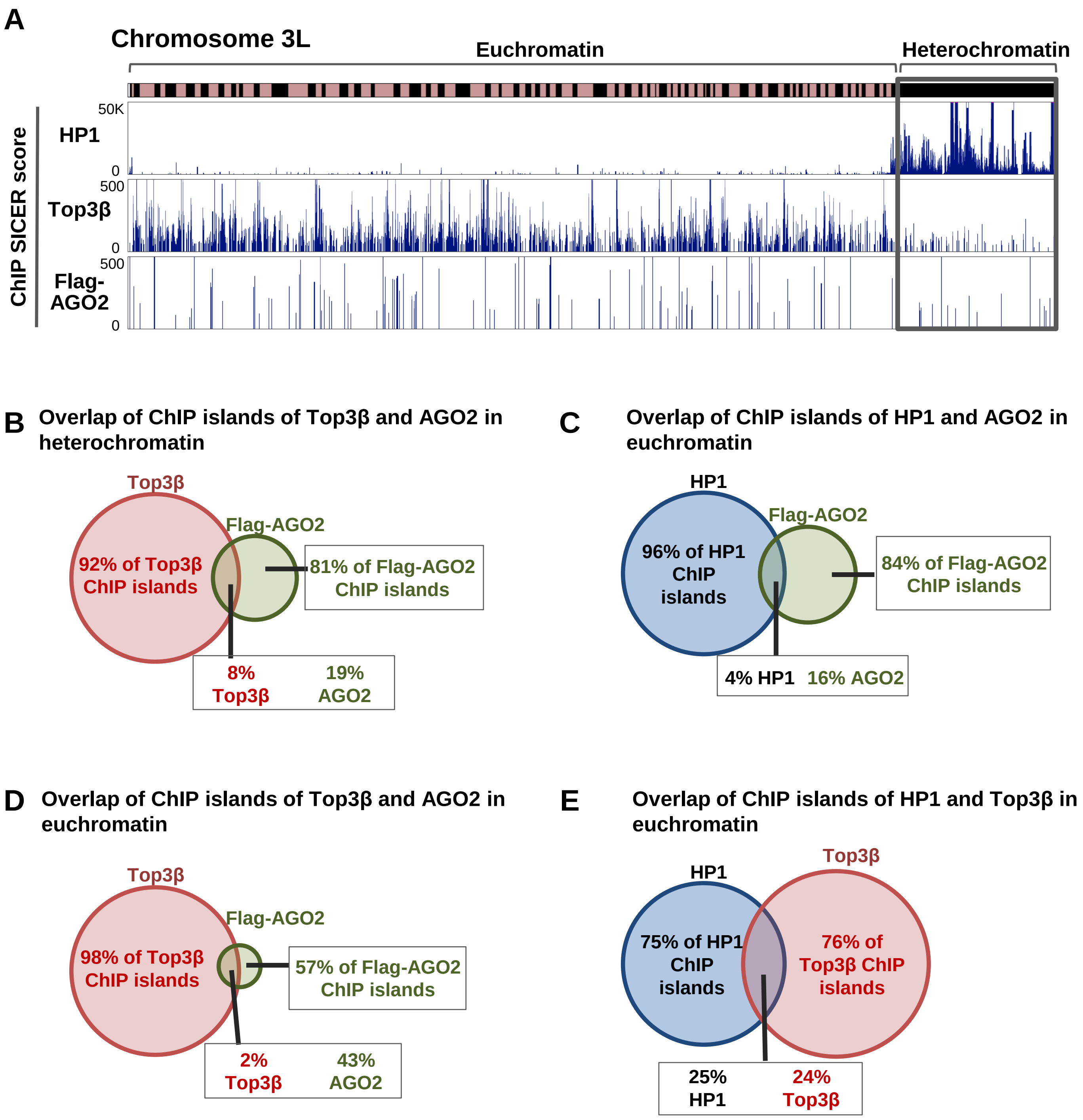

Supplementary Figure 6. A fraction of ChIP signals of Top3β and AGO2 overlap with each other and with those of HP1

(A) Bedgraphs show the comparison of ChIP SICER signals for HP1 (top), Top3β (middle) and Flag-AGO2 (bottom). It should noted that the Flag-Ago2 signals have subtracted the non-specific background, which was determined by Flag ChIP using w^{1118} control line that lacks expression of Flag proteins. The scale in HP1 is 100 fold higher (50,000) than the other two (500). The graph only shows chromosome 3L as an example.

(B) Venn diagrams showing the overlap of ChIP SICER islands between Top3β and Flag-AGO2 (B) in the pericentric heterochromatin of chromosome 2 and 3. The quantifications in **(B-E)** are based on the length of the SICER signals.

(C-E) Venn diagrams showing the overlap of ChIP SICER islands between HP1 and Flag-AGO2 (**C**), and HP1 and Top3β (**D**), and Top3β and Flag-AGO2 (**E**), in the euchromatin of chromosome 2 and 3.

Supplementary Figure 7

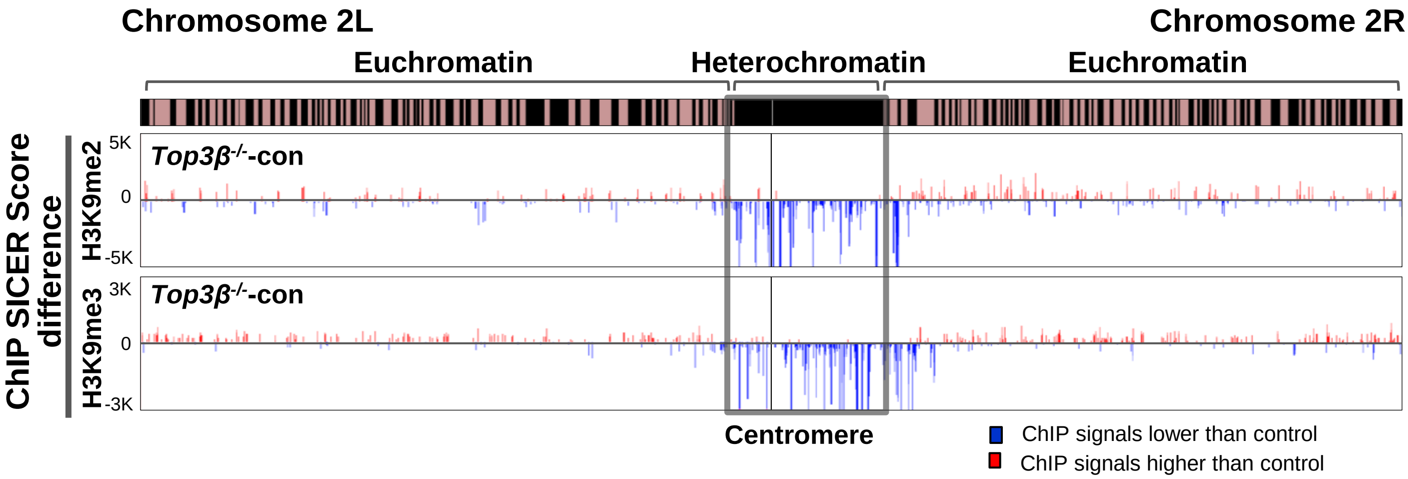

Supplementary Figure 7. Top3β promotes methylation of H3K9 in heterochromatin

Bedgraphs displaying SICER score difference (Y-axis) of H3K9me2 and H3K9me3 ChIP signals between the *Top3β* mutant and the control (*w*^{1118}) for chromosome 2. X-axis indicates chromosome locations of the islands on dm6 version of *Drosophila* genome. Only islands with signals higher than 200 with FDR<5% are shown. The islands with decreased and increased signals are marked with blue and red, respectively. The square in the center highlights pericentric heterochromatin and shows reduction of H3K9 methylation. The scale of the chromosome may not be accurate.

Supplementary Figure 8

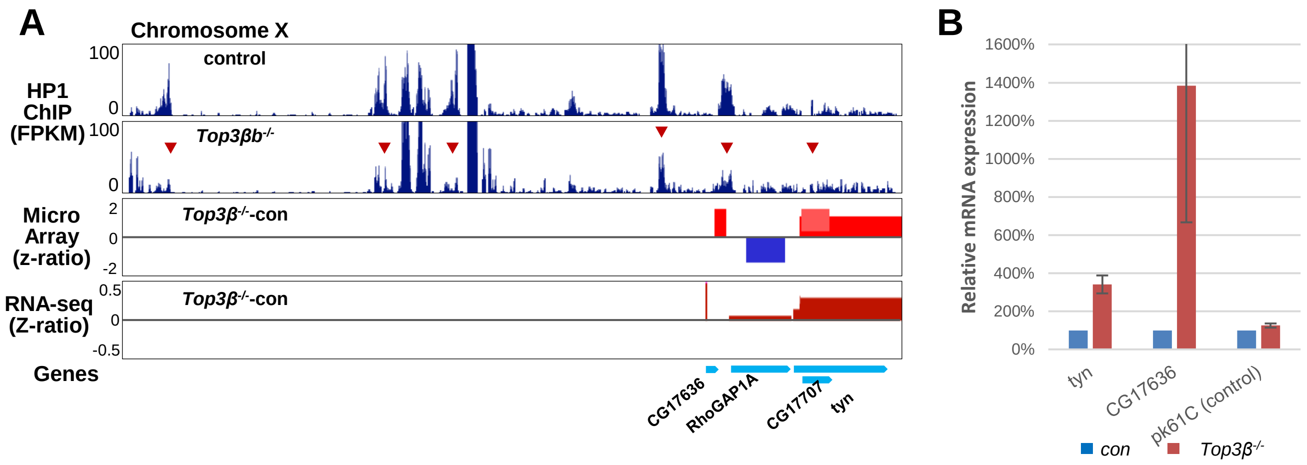

Supplementary Figure 8. Top3β is required for heterochromatin formation and transcriptional silencing

(A) Bedgraphs of ChIP-seq, cDNA microarray, and RNA-seq data show reduced HP1 signals and increased gene expression in sub-telomeric region of chromosome X. Arrows in the ChIP-seq bedgraph indicate the regions with reduced HP1 signals in *Top3β* mutant when compared to the control (*w*^{1118}). The bedgraphs of RNA-seq and cDNA microarray used Z-ratios (Y-axis) to show the difference in transcript levels between the *Top3β* mutant and control for genes in this region. The decreased and increased RNA signals in *Top3β* mutant are marked by blue and red, respectively. The chromosome locations of genes are indicated at the bottom graph.

(B) Graphs of RT-qPCR data show de-silencing of the two genes in chromosome X sub-telomere as listed in (A). *pk61c* served as a negative control. The error bars represent standard error.

Supplementary Figure 9

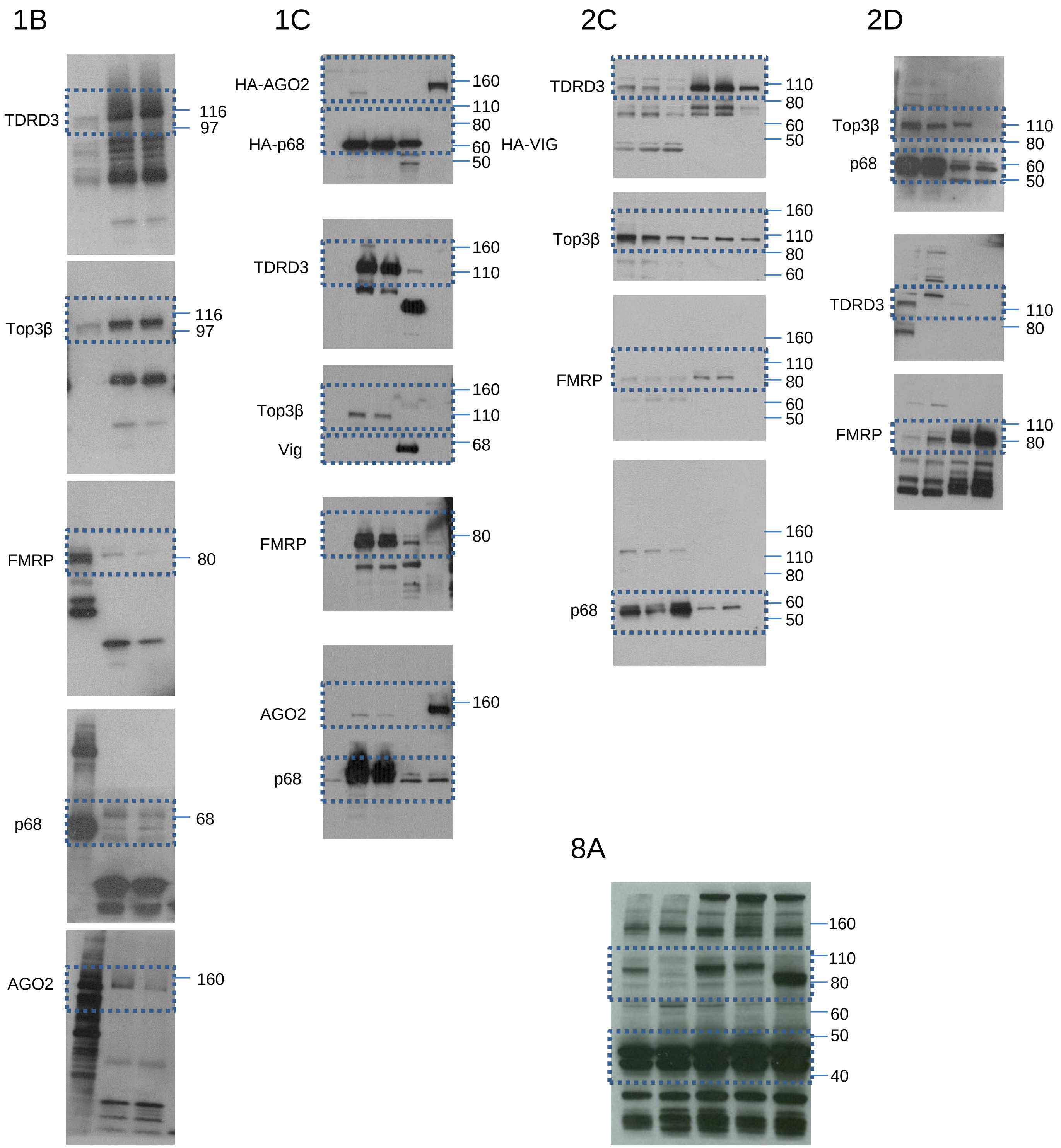

Supplementary Figure 9. Uncropped Western blot images

The scanned images of uncropped Western blot films for the main figures. The corresponding figure numbers are on shown the top left.
